# Designed Strategies for Fluorescence-Based Biosensors for the Detection of Mycotoxins

**DOI:** 10.3390/toxins10050197

**Published:** 2018-05-11

**Authors:** Atul Sharma, Reem Khan, Gaelle Catanante, Tauqir A. Sherazi, Sunil Bhand, Akhtar Hayat, Jean Louis Marty

**Affiliations:** 1 BAE: Biocapteurs-Analyses-Environnement, Universite de Perpignan Via Domitia, 52 Avenue Paul Alduy, 66860 Perpignan CEDEX, France; p20120407@goa.bits-pilani.ac.in (A.S.); kreemjadoon@gmail.com (R.K.); gaelle.catanante@univ-perp.fr (G.C.); 2 Biosensor Lab, Department of Chemistry, Birla Institute of Technology and Science, Pilani K. K. Birla Goa Campus, Zuarinagar, Goa 403726, India; sgbhand@gmail.com; 3 Department of Chemistry, COMSATS Institute of Information Technology, Abbottabad 22060, Pakistan; sherazi@ciit.net.pk; 4 Interdisciplinary Research Centre in Biomedical Materials (IRCBM), COMSATS Institute of Information Technology, Lahore 54000, Pakistan; 5 School of Pharmaceutical Sciences, MVN University-Palwal, Haryana-121105, India

**Keywords:** mycotoxins, fluorescence assay, biosensors, nanomaterials, fluorescence quenching, food samples

## Abstract

Small molecule toxins such as mycotoxins with low molecular weight are the most widely studied biological toxins. These biological toxins are responsible for food poisoning and have the potential to be used as biological warfare agents at the toxic dose. Due to the poisonous nature of mycotoxins, effective analysis techniques for quantifying their toxicity are indispensable. In this context, biosensors have been emerged as a powerful tool to monitors toxins at extremely low level. Recently, biosensors based on fluorescence detection have attained special interest with the incorporation of nanomaterials. This review paper will focus on the development of fluorescence-based biosensors for mycotoxin detection, with particular emphasis on their design as well as properties such as sensitivity and specificity. A number of these fluorescent biosensors have shown promising results in food samples for the detection of mycotoxins, suggesting their future potential for food applications.

## 1. Introduction

Mycotoxins are low molecular weight and thermally stable secondary metabolites of toxigenic molds that mainly belong to the genera: *Aspergillus*, *Penicillium*, *Alternaria*, and *Fusarium* [1,2]. Mycotoxins readily colonize crops and contaminate them at both pre and post harvesting level. For example, *Fusarium* and *Alternaria* produce toxic metabolites in the field while *Penicillium* and *Aspergillus* contaminate the food stuff during drying and storage processes. These toxins are present in the mycelium and spores of the toxic molds [3]. In the literature, approximately 100 different species of mycotoxins producing fungi have been reported, which can produce more than 400 toxigenic metabolites. The food regulatory authority (FAO) estimates that 25% of the global agricultural products are contaminated with mycotoxins which is a serious concern to public health and can trigger severe economic losses [4]. Prevalence and toxicities of mycotoxins—such as aflatoxins (AFs), ochratoxins (OTs), trichothecenes, patulin (PTL), citrinin (CIT), fumonisins, zearalenone (ZEA), deoxynivalenol (DON)—possess potential effect on human health and agronomic perspective. Mycotoxin analysis in food commodities and beverages including cereals, wheat, rye, maize, millet, cottonseeds, peanuts, milk, red wine, beer, coffee beans, cocoa, pistachio nuts, and many more dried or stored products is an important practice to ensure their quality and to eliminate the risk of consuming contaminated foods. Concentration of mycotoxins in such agricultural commodities depend upon the tropical conditions like moisture, high temperature, monsoons, flash floods, and unseasonal rains that could increase the fungal proliferation and consequently mycotoxins produced.

Mycotoxins are a class of ubiquitous cytotoxic chemicals that have severe hazardous effects on human and animal health due to their carcinogenic, mutagenic, teratogenic, nephrotoxic, estrogenic, and an immunosuppressive nature [4,5]. Owing to these acute and chronic toxicities, mycotoxins have potential to be used in bioterrorism. Evidence from history shows that mycotoxins have been extensively used by the Soviet Union as a biowarfare agent during the Cold War (1974–1981), resulting in high mortality [6,7].

### Regulatory Limits of Myctoxins

Food regulatory authorities—such as the World Health Organization (WHO), Food & Agriculture Organization of United Nations (FAO), and European Commission Directorate General for Health & Consumers—have set dietary limits for different mycotoxins in food stuff, e.g., maximum permissible limit for aflatoxins in food is 0–20 ng mL^−1^ and 0–50 ng mL^−1^ for humans and animals respectively. Similarly maximum permissible concentration for ochratoxin A (OTA), defined by European Union Commission Regulation is 5.0 ng mL^−1^ for raw cereals and 3.0 ng mL^−1^ for cereal-based products [8]. In maize and maize-derived food products, the permitted human consumption of fumonisins, i.e., FB1 and FB2, is regulated as 1000 ng mL^−1^ [9]. Most of the countries have set the maximum permissible limit for ZEA as 20–1000 ng mL^−1^ for raw and processed food products [8,10]. For corn and wheat, the specific limit of ZEA is 60 ng mL^−1^. Likewise, for DON, European Community (EC) declares the maximum tolerable concentration as 200 ng mL^−1^ for processed cereals and 1250 ng mL^−1^ for raw or unprocessed cereals [8]. In general, the countries worldwide follow the regulations set by European Union commission but few countries have assigned their own regulations for the limit of mycotoxins in food and feed. To follow these regulations, Comprehensive detection techniques to monitor the amount of toxins in food and feed are required.

## 2. Monitoring of Mycotoxins

As the chronic nature of mycotoxins have been recognized and certain permissible limits have already been established for these chemicals in edible stuff, now, major attention is paid towards the development of cost effective and efficient analytical tools for detection of mycotoxins in food commodities. The earliest method used for this purpose was thin layer chromatography (TLC) [11]. The advancement in analytical sciences also improved chromatographic detection methods. For example, HPLC coupled with different types of detectors—like ultra-violet (UV), gas chromatography coupled with mass spectroscopy (GC-MS) [12], and liquid chromatography coupled with mass spectroscopy (LC-MS) [13]—are also now recognized as highly sensitive techniques for mycotoxin analysis.

During the 1970 and 1980s, there was a remarkable invention of immunoaffinity-based detection techniques, exploiting antibody-antigen reactions. In a very short period of time, ELISA kits were used extensively as a delicate screening method for mycotoxins. Technological advancement further offered immuno-chromatographic test strips which were introduced as a screening tool in safety field. Although, strip tests are easy to perform but these are semi-quantitative and do not depict a full picture of contaminants. All these above mentioned techniques have advanced features and each offer unique advantages, but these techniques also suffer from inherent limitations such as involvement of complicated instrumentation, thus restricting their practical application and making on-site utility practically impossible, these are time consuming and are not user-friendly, requiring skilled personnel to perform the analysis, and high running costs.

Alternatively, biosensors have emerged as the most promising tool and meet most of the requirements of an efficient analytical device for mycotoxins monitoring. On the basis of different types of transducer material, biosensors may be electrochemical, optical, piezoelectric, and calorimetric. Among all these classes of biosensors, optical biosensors have significant advantages over the others because of their simplicity, sensitivity, and specificity. Optical biosensors are further classified into sub-classes like colorimetric, fluorescence, phosphorescence, reflection, refraction, surface plasmon resonance, resonance dispersion, Raman scattering, infrared absorption, and chemiluminescence biosensors, among which fluorescence biosensors have recently been the most exploited group of biosensors.

## 3. Fluorescence Biosensors

Fluorescence-based biosensors have been explored for various applications such as medical diagnostics, drug delivery, drug discovery, environmental monitoring, and food safety. Keeping the basic principles in view, various working strategies can be designed for fluorescence biosensors to detect different analytes. Numerous parameters can be explored in fluorescence biosensors such as fluorescence intensity, fluorescence anisotropy, decay time, energy transfer (radiative or non-radiative), quenching efficiency, and quantum yield.

### 3.1. Formats in Fluorescence Biosensors

Many molecules exhibit fluorescence naturally, i.e., in one state they are fluorescent and another non-fluorescent. By using this property, a very simple fluorescence biosensor can be designed, e.g., NADH is fluorescent while NAD^+^ is not. Therefore, all the enzymatic reactions based on NAD/NADH can be subjected to fluorescence-based detection. This scheme is widely used by analytical chemists to detect and quantify various analytes. This is direct fluorescence format. Similarly, many proteins and other biomolecules (nucleic acids, NADH, Flavin nucleotides green fluorescent proteins) have intrinsic fluorescence characteristics and, upon binding with ligands or when ligands bind with these proteins, a change in the fluorescent behavior of these molecules occurs either in terms of emission intensity, polarization, etc.

In contrast, most of the analytes are non-fluorescent. Thus, to make them detectable by fluorescence spectroscopy, different fluorescence labels or probes are used in the course of action. Labels are attached to the analyte of interest by a covalent interaction through any reactive group such as hydroxyl, carboxyl, amino, or sulfhydryl groups that assist in establishing a chemical linkage between the species of interest and the label. In general, labels, probes, or tags are relatively small size agents consists specific functional group with intrinsic fluorescent character that render the detectable sensitivity to the molecule at which they are attached (nucleic acids, proteins or any other molecules). The label and probe differs each other in term of their response to the environment, such that the label is just covalently attached to the analyte and does not interfere with other chemical species in the environment, but the fluorescent probe is not supposed to be inert and is highly responsive to elements of the micro environment such as pH, ions, or variation in oxygen content. In the context of fluorescence biosensors, aptamers are excellent candidates as a bio-recognition element because of their easy and straightforward modification along with high specificity and selectivity. Unlike other biological elements (antibodies, enzymes, peptides), aptamers offer great flexibility for their chemical modification. Hence, a lot of aptasensor formats can be designed for a wide range of analytes.

#### Fluorescence-Based Aptasensors

One of the most commonly used formats among fluorescence aptasensors is the use of aptabeacons, which are a modified version of the traditional molecular beacon. Similar to the molecular beacon, aptabeacons have a hairpin like structure end, which is labeled with a fluorophore and a quencher. When the target molecule binds with the aptamer, the binding of analyte disturbs the initial conformation of Forster resonance energy transfer (FRET) pair, causing the fluorescence signal to turn-on [14].

Another type of biosensor named ‘aptamer switched probe’ is designed in which an aptamer and a short DNA strand complementary to small part of aptamer attached with polyethylene glycol (PEG) linker. In this strategy, a FRET pair (a fluorophore and a quencher) is attached to the termini of DNA strand in such a way that fluorescence is completely quenched. In the absence of analyte, the complementary DNA hybridized with the aptamer keeping the fluorophore and quencher in close proximity that results in quenching of fluorescence. As the target molecule approaches the aptamer, the hybridization of a probe with an analyte disturbs the complementary DNA hybridization and hence displaces the quencher resulting in the recovery of fluorescence [15]. Several other biosensors also work on the same principle [16,17].

Another commonly designed biosensor is based on pyrene dye. Pyrene monomer exhibits very low fluorescence intensity but, when two monomeric units come in close proximity with each other, they result in pyrene eximer formation which has a long fluorescence lifetime (approx 40 nanoseconds) and a long Stokes shift. Most of the chromophores exhibit a fluorescence lifetime of less than 10 nano-seconds. This property of pyrene is efficiently exploited in construction of different biosensor for a lot of analytes.

The selection of a label also defines the working principle of the biosensor such that some sensors operate through FRET phenomenon or sometimes quenching of the fluorescence is measured to quantify the analyte.

### 3.2. Criteria for Selection of a Fluorescent Label

Before selection of a fluorescent probe, some important considerations must be taken into account while designing an appropriate label for a particular system, such as the label must be conveniently excitable without the excitation of other components present in the matrix. It must be able to produce a clearly detectable signal and should have a high molar absorption coefficient and high fluorescent quantum yield. It should have higher stability and solubility in the medium and contain some specific functional groups to aid in the site-specific labeling. In case organic dyes are used as a fluorescent tag, selection should be made on the basis of parameters such as; high molar absorbance (*ε*) and quantum yield. Sensing strategies that are based on the analysis or calculation of fluorescence intensity as a result of quenching mainly depend upon the variation in quantum yield. Thus, molar absorbance and quantum yield of a dye should be as high as possible.

### 3.3. Types of Fluorescence Labels

#### 3.3.1. Organic Dyes

Organic dyes are widely used as tags in fluorescence sensing. They are easily available, low cost, and the most versatile class of fluorescence reporters. The number of natural and synthetic organic fluorophores is so great that a researcher can easily pick a tag from the pool of dyes that is suitable to his requirements in terms of chemical reactivity and spectroscopic properties. Sensing technology is the major area of application of these dyes. Owing to their advantages in sensors and other fields, the number of synthetic dyes grows exponentially. Most commonly-used fluorescent dyes are based on cyanine structure or xanthene dyes. Fluorescein and rhodamine are the first organic dyes used for fluorescent tagging. Despite their huge advantages, these dyes suffer from some disadvantages like pH sensitivity, photo-bleaching, and hydrophobicity.

#### 3.3.2. Nanomaterials as Fluorophores and Quenchers

To overcome technical obstacles of organic dyes, nanomaterials—like semiconductor quantum dots [18,19], upconversion nanoparticles [20], and organic polymers nanoparticles—have been explored as excellent alternatives [21].

Quantum dots (QDs) are efficient signal generating nano-probes. These are semiconductor nanocrystals of <10 nm in size and composed of elements belonging to groups II and VI (Zn, Te, Se, Cd) or group III-V (P, As, In). Because of their nano size, strong quantum confinement effect results in broad absorption band in UV–vis region and narrow emission spectra with tunable optical properties. The optical properties of QDs mainly depends on the constituent material, particle size, disparity (size distribution), type of QDs (core or core-shell quantum dots), and surface chemistry (type of material used for surface passivation). Thus, by manipulating these parameters, one can easily obtain desired optical properties for a specified application [22].

Silica nanoparticles (Si-NPs) are another class of nanomaterials that has been extensively exploited in fluorescent biosensors. Si-NPs can serve dual function; one is to use silica NPs as a solid support in FRET assay for smooth material handling [23,24] and secondly they can be used to doped with different fluorescent dyes for signal enhancement. Dye doped silica nanoparticles have been used in the detection of nucleic acids, proteins, and pathogens [25,26,27]. Major advantages of using silica NPs in fluorescence biosensors are abundant and non-toxic nature of silicon, ease of conjugation with biomolecules due to high surface to volume ratio of NPs, and that a large number of dye molecules can be incorporated inside the nanoparticles [28].

Au-NPs are one of the best FRET-based quenchers due to their outstanding optical properties [18,29]. Moreover, Au-NPs are resistant to photobleaching and have stable signal intensities. In FRET-based assays Au-NPs are frequently used as FRET acceptor in combination with fluorescent dye as donor. One of the simplest and widely used formats of this kind is the use of Au-NPs in molecular beacons and aptabeacons [30,31].

Silver nanomaterials (nanoparticles and nanaoclusters) are also efficient FRET acceptors. In a fluorescence assay, these can serve a dual function as a FRET acceptor (quencher) as well as providing a supporting surface for bioassay and enhancing the assay performance. A 21-fold higher FRET rate constant is observed in the assays, where AgNPs are used as the supporting surface [32]. 

Carbon nanotubes (single walled and multi-walled), grapheme, and nanodimonds are mostly exploited in fabrication of optical biosensors to obtain quick and reliable responses [20,29,33]. In fluorescence biosensors, carbon nanotubes (CNTs), and graphene are used as quenchers of many dyes in FRET-based assays. π-π stacking effect between the nucleotide bases of dye labeled single-stranded DNA/RNA and CNTs brings the dye into close proximity of CNTs resulting in turn off fluorescence signal. Binding of analyte disturbs this π-π stacked structure and results in recovery of fluorescence. Other nanomaterials used in fluorescence-based biosensors are magnetic nanoparticles, nanoceria, and upconversion nanoparticles [34].

## 4. Fluorescence-based Biosensors for Mycotoxins Analysis

### 4.1. Immunosensing Platforms 

#### 4.1.1. Ochratoxin A (OTA) Detection

**Figure 1:**
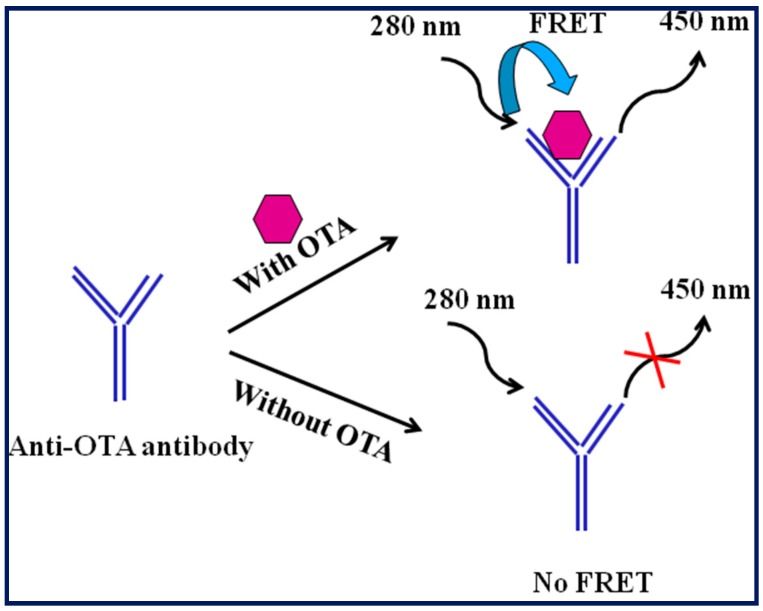
A label-free, direct, and non-competitive homogeneous Fluorescence resonance energy transfer (FRET) immunoassay system for ochratoxin A (OTA) detection.

In the 20th century, OTA determination using immunosensors have gained significant interest [35]. Initially, a fluorescence polarization competitive immunoassay (FPIA) method based on monoclonal antibody was developed for OTA determination. The developed assay was able to OTA in the concentration range of 5.0 × 10^3^–200.0 × 10^3^ ng mL^−1^, with a detection limit of 3.0 × 10^3^ ng mL^−1^ [36]. When FPIA method was applied to barley samples spiked with 50.0–500.0 × 10^3^ ng mL^−1^ OTA, recoveries were higher than 90% compared to an indirect competitive ELISA. Moreover, the analysis of naturally contaminated barley samples showed some disagreements between the results obtained among the two techniques due to a stronger matrix effect observed with ELISA. Ngundi et al. has reported the development of a rapid and highly sensitive competitive immunoassay for the detection and quantification of OTA [37]. OTA quantification was based on the formation of fluorescent immunocomplex on the waveguide surface. The developed assay was able to detect OTA as low as 0.1 ng mL^−1^ OTA in cornmeal. Later on, the same group also reported the development of fluorescence-based multiplexed competitive assay for the simultaneous detection of OTA and deoxynivalenol (DON) [38]. The limits of detection (LODs) were reported as 15 and 150 ng mL^−1^ for OTA and DON, respectively. Based on a monoclonal antibody and an ochratoxin A (OTA)-fluorescein tracer, a FPIA based platform was demonstrated for rapid screening of OTA in red wine [39]. The assay exhibited a LOD of 0.7 ng mL^−1^ OTA with analysis in less than 10 min. Fluorescence resonance energy transfer (FRET) is a widely used tool for investigating a number of molecular interactions. As depicted in Figure 1, a label-free, direct, and noncompetitive homogeneous FRET-based immunosensing platform where OTA coupled with the anti-OTA antibody participates in FRET phenomenon was developed for the OTA detection with great specificity and a LOD of 1.0 ng mL^−1^ OTA. Performance of the developed assay platform was also verified for OTA detection in wheat sample [40]. Another study has reported the label-free homogeneous fluorescence immunoassay for OTA detection based on a fluorescence change of OTA in the OTA/antiOTA complex. The system was relying on generation of fluorescence from the dianionic form of OTA in the OTA/anti-OTA complex. Under optimized experimental conditions, the fluorescence immunoassay was able to detect OTA in a highly specific manner with a LOD of 5 × 10^−4^ ng mL^−1^ OTA. Later on, a comparative fluorescence-based and flow-based immunosensing platforms for the analysis of OTA were reported [41]. The theoretical lowest LOD enabled by affinity of the anti-OTA antibody was 12 × 10^−4^ ng mL^−1^ (considered as 80% binding). No significant matrix interference was reported during wine and cereal samples analysis. An automatic solid-phase extraction (SPE) system coupled with a fluorescence detector was developed for the sensitive determination of OTA, which was able to detect OTA in wheat samples in the range 3–18 ng mL^−1^ (LOD 1.2 ng mL^−1^ OTA). Dynamic binding capacity of dry MIP particles for 45 mg was calculated as 118 ± 9 ng of OTA (*n* = 3) [42]. Liang et al. (2016) reported a novel fluorescence coupled ELISA to detect OTA by using the glucose oxidase (GOx)-mediated fluorescence quenching of mercaptopropionic acid-capped cadmium telluride (CdTe) quantum dots (MPA-QDs) [43]. 

In this study, glucose oxidase (GOx) was used as an alternative to HRP (horseradish peroxidase) for the oxidization of glucose into hydrogen peroxide (H_2_O_2_) and gluconic acid. The mercaptopropionic acid MPA-QDs were employed as a fluorescent signal output, whose fluorescence variation was extremely sensitive to the presence of H_2_O_2_ or hydrogen ions in the solution. The presented platform demonstrated a good linear detection 0.0024–0.625 ng mL^−1^ with a LOD of 0.0022 ng mL^−1^ OTA in corn extract. Similarly, an improved competitive fluorescent-ELISA for the ultrasensitive detection of OTA using hydrogen peroxide (H_2_O_2_)-induced fluorescence quenching of mercaptopropionic acid-modified CdTe quantum dots (QDs) was also reported [44]. Herein, the catalase (CAT) was labeled with OTA as a competitive antigen to connect the fluorescence signals of the QDs corresponding to concentration of target analyte. The developed platform was able to detect OTA ranging from 5 × 10^−5^–1 × 10^−4^ ng mL^−1^ with a LOD of 5 × 10^−5^ ng mL^−1^ and IC_50_ 53 × 10^−5^ ng mL^−1^. A silver nanoparticles (AgNPs)-based fluorescence-quenching competitive coupled with lateral flow immunoassay (cLFIA) was developed. The cLFIA platform exhibited a LOD of 0.06 ng mL^−1^ OTA in wine [45]. Different fluorescence-based immunosensors for OTA detection have been summarized in Table 1.

#### 4.1.2. Aflatoxins Detection

Due to occurrence of AFs in various food and feed products, there is an immense need for AF analysis. A platform based on surface plasmon-enhanced fluorescence spectroscopy (SPFS) for detection of aflatoxin M1 (AFM1) in milk was reported [46]. In SPFS, the binding of fluorophore-labeled or analyte molecules to the sensor surface was probed with surface plasmons (SPs), where the emitted fluorescence light was detected. The developed sensor allowed the detection of AFM1 in milk within 53 min with a LOD of 6 × 10^−4^ ng mL^−1^. A competitive immunoassay for sensitive determination of AFB1 was also reported [47]. In this strategy, the nanomaterial doped (rhodamine B i.e., RB as fluorophore) silica nanoparticles were used as support for immobilization of monoclonal anti-AFB1 antibodies. Developed platform showed narrower dynamic range of 0.5–7 ng mL^−1^ and 0.5–30 ng mL^−1^ with LODs of 0.2 and 0.1 ng mL^−1^ AFB1 with the flow setup compared to the microtiter plate (MTP) format. Use of labeled and non-labelled nanomaterial in small molecule determination has also gained significant attention due to the application of quantum dots (QDs) in aflatoxin analysis. Several FRET-based immunosensors were reported for highly sensitive detection of AFB1 employing the different-sized quantum dots [48,49]. Molecular interaction of anti-AFB1 mAbs and AFB1 promoted one or more acceptors bound with a multivalent AFB_1_-labeled donor, resulting in FRET from the green to red QDs. Under optimized experimental conditions, immunosensor exhibited the detection range in logarithmic scale over 0.06–5 ng mL^−1^ (0.19–16 pM) and an LOD of 0.04 ng mL^−1^ (0.13 pM) in rice extracts. Obtained recoveries were from 83.27–97.36% for inter- and intra-assay measurements. Zhang et al. (2015) reported the development of a portable immunosensor based on chromatographic time-resolved fluorescence (TRF) for on-site determination of AFB1 in food and feed samples [50]. The developed immunosensor showed a magnified positive signal and low signal-to-noise ratio in time-resolved mode, which was due to the absence of noise interference caused by excitation light sources. This platform demonstrated a wider dynamic range of 0.2–60 ng mL^−1^ with LODs from 0.06 to 0.12 ng mL^−1^ and recovery from 80.5 to 116.7% for different food and feed sample matrices. In the meantime, a fast and easily performed fluorescence polarization immunoassay (FPIA) for AFB1 beer sample was also reported. Condition for sample pretreatment techniques were optimized and found to be suitable for beer sample analysis. The LOD was found to be 1 ng mL^−1^ with apparent recovery values of 89–114% for lager and 80–125% for dark beer sample [51]. Multiplex planar waveguide fluorescence immunosensor (MPWFI) achieving AFM1 detection by applying the principle of immunoreactions and total internal reflect fluorescent (TIRF) was also reported [52]. The immunosensor exhibited a working range of 0.073–0.400 ng mL^−1^ AFM1 and a LOD of 0.045 ng mL^−1^ providing no significant interference in presence of melamine in milk sample. A simple, low-cost, and reusable immunosensor for ultrasensitive detection of AFM1 using a portable evanescent wave-based optofluidic biosensing platform (EOBP) was developed by Lou et al. (2016) [53]. The effects of several organic solvents on the Ag-Ab binding reaction in heterogeneous and homogeneous solutions were evaluated and found to be more significant on homogeneous binding over heterogeneous binding reaction. The detected fluorescence signal by EOBP was linear to AFM1 concentration with a regeneration capability of 200 times and a LOD of 0.005 ng mL^−1^. 

#### 4.1.3. Other Mycotoxins Detection

A fiber-optic immunosensor constructed by covalently immobilized mAb-FB1 through a heterobifunctional silane was developed to measure the FB1 [54]. The immunosensor exhibited a working range of 10–1000 ng mL^−1^ FB1 with an IC_50_ 70 ng mL^−1^ and a LOD of 10 ng mL^−1^. The sensor showed cross-reactivity with FB2 but did not react with hydrolyzed FB1, sphinganine, or tricarballylic acid. A sensitive flow-through based immunosensor for detection of zearalenone in cereal samples has also been described [55]. The immunosensor exhibited an IC_50_ value of 0.087 ng mL^−1^ and dynamic range 0.019–0.422 ng mL^−1^ and a LOD of 0.007 ng mL^−1^. For simultaneous determination of FB_1_ and FB_2_ in maize, A FPIA platform was developed. The FPIA employing the both FB_1_-FITC and mAb 4B9 showing 98.9% cross-reactivity (CR) toward FB_2_ was used for simultaneous detection of FB_1_ and FB_2_. After optimization, the FPIA revealed a LOD of 157.4 ng mL^−1^ for FB_1_ and 290.6 ng mL^−1^ for FB_2_, respectively with total time needed for FPIA including sample pretreatment was <30 min [56]. Beloglazova et al. (2014) developed the novel multiplex fluorescent immunoassays based on quantum dot nanolabels for simultaneous determination of several mycotoxins such as DON, ZEN, AFB1, T2-toxin, and FB1. The reported LODs for simultaneous determination of DON, ZEN, AFB1, T2-toxin, and FB1 by SAM- FLISA were 3.2, 0.6, 0.2, 10, and 0.4 ng mL^−1^, respectively, whereas for the double analyte multiplex (DAM) FLISA, 1.8 and 1 ng mL^−1^ were found for ZEN and AFB1 respectively [57]. Various immunosensing platforms for mycotoxins other than OTA have been tabulated in Table 2.

### 4.2. Aptamer-Based Assays

#### 4.2.1. OTA Detection

With the advancements in nanotechnology and synthetic receptor chemistry, the integration of fluorescent labels and aptamer/DNA, researchers have developed several fluorescence-based structure switching aptasensor or aptamer assays [58,59]. Sheng et al. (2011) developed an aptasensing platform where a fluorescein modified aptamer (FAM-modified) was adsorbed onto the basal plane of graphene oxide (GOx) via the π-π stacking force resulting in the quenching of fluorescence signal via energy transfer from dye to GOx [60]. Whereas, in the presence of target analyte (OTA), the target induced conformational changes of the aptamer, leading to the formation of an antiparallel-G-quadruplex. In principle, the increase in fluorescence intensity corresponding to the OTA concentration was measured. The presented method showed a linear detection range from 8.01 × 10^−1^–14.133 × 10^−3^ ng mL^−1^ with a LOD of 0.767 ng mL^−1^ on bare GOx, and 0.955 ng mL^−1^ on PVP-modified GOx surface.

**Figure 2:**
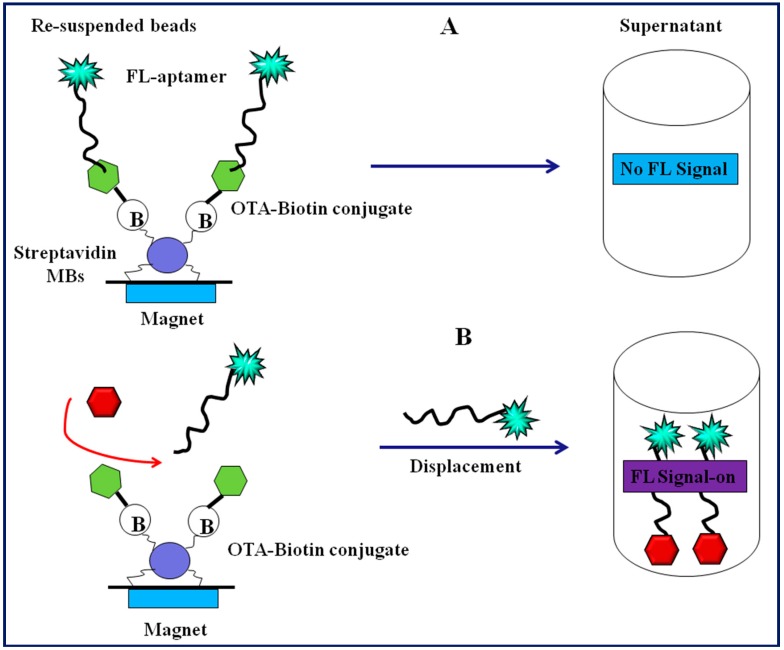
The assay principle for the fluorescence detection methodologies (**A**) displacement assay; (**B**) competition assay.

Later on, utilizing single-walled carbon nanotubes (SWCNTs) as fluorescence quencher, a fluorescent aptasensor for sensitive and selective determination of OTA was constructed by Guo et al. [61]. Compared to GOx-based sensor, the SWCNT-based aptasensor exhibited a LOD of 9.73 ng mL^−1^ with detection range of 10.09–80.76 ng mL^−1^. Similarly, Duan et al. established a new structure switchable fluorescent aptasensor for OTA detection [62]. The presented method employed the fact that when the immobilized aptamer specific to OTA binds with the target OTA it induces the conformation change in the aptamer. These conformational change results in the dissociation of the FAM-labelled complementary DNA chain from aptamer, leading to the fluorescent signal change (fluorescence on). Under optimized conditions, the method was able to detect OTA in the linear range of 0.002–10 ng mL^−1^ with a detection limit of 0.001 ng mL^−1^. The authors also validated the feasibility of method in corn samples. Later on, Chen et al. designed a simple and rapid method for OTA detection based on target induced structure switching signaling aptamer [63]. The method was based on the universal principle of target induced conformational change from aptamer/DNA duplex to target-aptamer duplex complex, which led to the release of a hybridized quencher-labelled DNA chain from the FAM-labeled OTA aptamer, generating a substantially increased fluorescence intensity. Under optimized assay conditions, the assay exhibited a wide linear detection range of 1–100 ng mL^−1^ with a LOD down to 0.8 ng mL^−1^ and an analysis time of less than 1 min. Additionally, the designed aptamer assay exhibited high selectivity and specificity, and no significant interference against structural analogues was observed. Various nanomaterials (gold, terbarium, carbon nanotubes) as fluorescence quenchers have been explored for the development of aptasensing platform to detect OTA [64,65,66]. Chen et al. developed a signal-on fluorescent aptasensor for OTA detection based on fluorescent DNA-scaffolded silver-nanocluster (AgNCs), structure-switching of anti-OTA aptamer (Ap) and magnetic beads (MBs) [67]. The feasibility of method for detecting OTA in real samples of wheat was also demonstrated. The method exhibited superior sensitivity with a detection limit as low as 0.002 ng mL^−1^ OTA with high specificity. Based on the dye molecule intercalates with DNA strands, Mckeague et al. designed a label free aptasensing platform for OTA detection based on intercalation of SYBR Green I. This label-free platform offered a rapid, selective, and sensitive OTA quantification with a LOD of 3.63 ng mL^−1^ and linear of quantification (LOQ) up to 40.38 ng mL^−1^ [68]. A generalized fluorescence-based aptasensing design employing carboxy- modified fluorescent (CMF) particles as a signal-generating probe and magnetic particles as a solid separation support was reported by Hayat et al. [69]. As depicted in Figure 2, as proof of concept, the proposed assay was used for OTA detection based on the displacement and competition format. The competition-based assays showed improved analytical characteristics as compared to the displacement assay. The competitive fluorescent aptamer assays exhibited high sensitivity with a detection limit and IC_50_ of 0.002 and 2.907 ng mL^−1^, respectively.

Recently, our group has designed and established two aptasensing platforms for OTA detection utilizing nanosurface impact of titanium dioxide nanoparticles (TiO_2_-NPs) [70,71]. In the first designed strategy, upon the non-covalent adsorption of FAM-labelled anti-OTA aptamer on TiO_2_ surface, the fluorescence of FAM-labeled aptamer was quenched. When OTA interacted with the aptamer, it induced the formation of aptamer-G-quadruplex complex, hence weakening the interaction between FAM-labeled aptamers and TiO_2_, resulting in fluorescence recovery. Under optimized conditions, the method showed a detection limit of 0.60 ng mL^−1^ with a good linearity in the range of 0.60–403.8 ng mL^−1^ for OTA. In second approach, a fluorescence aptaswitch was designed based on the aptamer modulated nano surface impact on the fluorescence particles. In this strategy, aptaswitch capitalizes on the surface chemistry of TiO_2_-NPs to quench the fluorescence of fluorophore carboxylate modified (FCM) particles, eliminating the need of bioconjugation with fluorophore. For practical application, the utility of designed platform was investigated for OTA as model analyte.

Use of quantum dots in small molecule detection has recieved remarkable attention. Recently, graphene and cadmium telluride (CdTe) quantum dots were reported for the development of fluorescence-based structure switching aptasensing platform for detection of OTA [19,72]. The developed platforms showed OTA concentration-dependent restoration of the fluorescence intensity with detection limit down to the ng mL^−1^ or pg mL^−1^. In the same context, a FRET-based fluorescence method for quick detection of OTA in agricultural products (e.g., flour and beer) was reported by Wang et al. [73]. The method was based on the use of highly fluorescent nitrogen doped carbon dots (CD) as energy donor and the DNA immobilized thiolated Ag-NPs as energy acceptor in the FRET system. Herein, the OTA can be detected in a concentration range from 4.04–2020 ng mL^−1^ with a LOD of 3.73 ng mL^−1^. Reported aptasensors based on fluorescence signaling for OTA detection have been summarized in Table 3.

#### 4.2.2. Aflatoxin and Other Mycotoxin Detection

**Figure 3:**
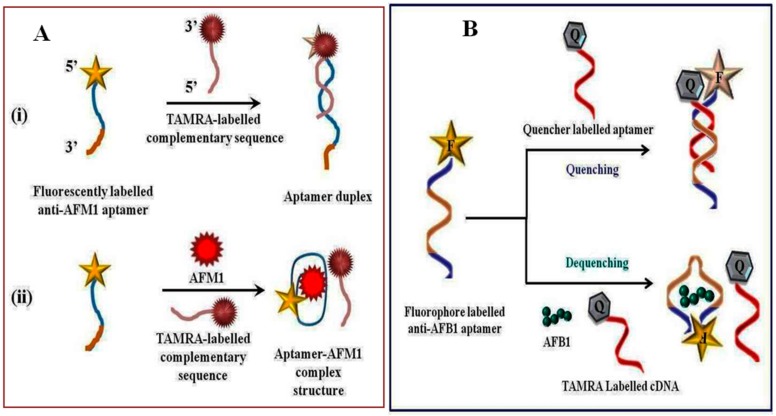
Structure switching fluorescence signaling platform for detection of (**A**) AFM1 and (**B**) AFB1. TAMRA: Carboxytetramethylrhodamine.

The development of structure switching assays based on fluorescence signal generation for aflatoxins detection are mainly based on the same format as those described for OTA detection. Nanomaterials are mainly employed as fluorescence quenchers. For example, our group has recently synthesized a composite of TiO_2_ and multiwalled carbon nanotubes to design a fluorescence quenching-based aptasensor for the detection of AFB1 [74]. An aptamer assay based on fluorescence recovery using a fluorescence quencher system composed of quantum dots and GOx was reported for detection of AFB1 [75]. To design the platform, a thiolated aptamer specific to AFB1 was linked to the surface of Q-dots via ligand exchange and the fluorescence of the aptamer modified-Q-dots was strongly quenched by GOx, which recovered on addition of AFB1. The proposed platform was evaluated both in phosphate buffer solution and in peanut oil. In peanut oil, the dynamic range was from 4.99 × 10^−1^–49.96 × 10^−4^ ng mL^−1^ and a detection limit of 0.44 ng mL^−1^ was obtained. Recently, Sharma et al. (2015) designed a structure switching signaling fluorescence aptamer assay for AFM1 detection [76]. Determination of AFM1 was based on the quenching–dequenching mechanism, where the hybridization of FAM-labelled anti-AFM1 aptamer (F-aptamer) with carboxytetramethylrhodamine (TAMRA) labelled complementary sequences (Q-aptamer) brought the fluorophore and the quencher into close proximity, resulting in maximum fluorescence quenching (Figure 3A). Under optimized experimental conditions, the developed method showed good linearity with limit of detection of 0.005 ng mL^−1^ for AFM1. Good recoveries were obtained in the range from 94.40% to 95.28% (*n* = 3) for AFM1 spiked milk sample providing no significant interference against AFB1 and OTA. Similarly, our group has constructed TAMRA quenching-based aptasensing platform for the detection of AFB1 [77], as depicted in Figure 3B. In this platform, we have compared the analytical performance of two aptamer sequences: seqA and seqB. The designed strategy exhibited good sensitivity and selectivity with a LOD of 0.2 ng mL^−1^ and a linear range from 0.25 to 32 ng mL^−1^. The aptasensor’s performance was tested in beer and wine samples and obtained recovery were in good agreement. In the same direction, two fluorescent aptasensors based on quantum dots—i.e., fluorescent nitrogen-doped carbon dots on gold nanoparticles (Wang et al.) and cysteamine-capped CdS quantum dots as a fluorescence probe (Tayebi et al.)—were designed and evaluated for detection of AFB1 and total aflatoxins, respectively [29,78]. 

**Figure 4:**
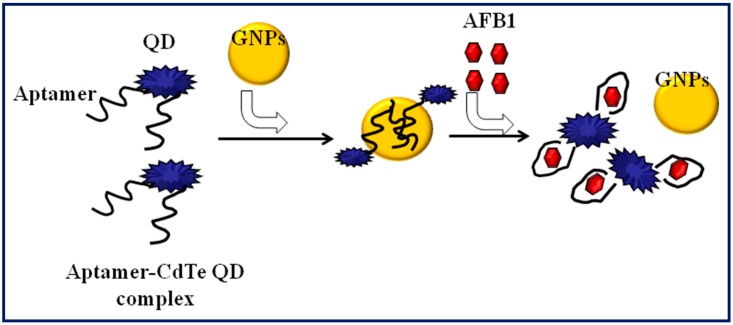
FRET-based nanoaptasensor using cadmium telluride (CdTe) quantam dots GNPs: Gold nanoparticles.

Sabet et al. developed a FRET-based aptasensor for selective and sensitive detection of aflatoxin B1 in peanut and rice by using aptamer-conjugated fluorescent Quantum dots (QDs) and Au-NPs as fluorescence quencher (Figure 4) [79]. The aptasensor exhibited a detection limit of 1.06 ng mL^−1^ with a linear range of 3.122–124.91 ng ml^−1^, and the method was successfully employed for AFB1 analysis in rice and peanut samples. Chen et al. reported the structure switching fluorescent aptamer assay for AFB1 detection in infant rice cereals [80]. Under optimized conditions, this assay was able to detect AFB1 down to the 1.6 ng mL^−1^ and exhibited linear response of 5–100 ng mL^−1^ AFB1, with percentage recovery in the range of 93.0–106.8%. Mukherjee et al. evaluated the performance of a competitive fluorescent aptasensor comparing with HPLC for detection of AFB1 [81]. This aptamer assay was also validated in food samples such as dried red chilies, groundnut, and whole pepper, and the obtained percentage recoveries were in the range of 92 to 102% at 10 ng mL^−1^ and 0.1 ng mL^−1^ levels.

An aptasensing platform based on multiplexed-FRET between multicolor upconversion fluorescent nanoparticles (UCNPs) as donors and Gox as the entire and effective acceptor was recently reported [20]. Due to the strong π-π stacking between the nucleobases of the aptamers and the sp^2^ hybridized atoms of GO, the aptamer modified-UCNPs can come in close proximity to the GO surface. In this arrangement, the strong upconversion fluorescence was completely quenched by the Gox due to the good overlap between the fluorescence emission of multicolor UCNPs and the absorption spectrum of Gox. The aptasensor provided a linear range from 0.1 to 500 ng mL^−1^ and a detection limit of 0.1 ng mL^−1^ FB_1_. A versatile and cost-effective aptamer assay based on fluorescence quenching principle was described by Goud et al. for the detection of the zearalenone (ZEN) [82]. In this platform, the exfoliated functional graphene oxide (FGOx) possessing high water-dispersibility was selected as an effective fluorescence quencher toward the fluorescence of FAM. It was observed that the FGO has more efficient quenching abilities than non-functionalized GOx. FGO-based assay allowed ZEN to be determined in the concentration range from 0.5–64 ng mL^−1^ with a LOD of 0.5 ng mL^−1^. Performance of methods was also successfully investigated to detect ZEN in spiked alcoholic beverage samples (beer and wine) and observed recovery values were in the range of 87 to 96% for ZEN spiked samples. The comparative studies on fluorescence-based aptasensing platforms for aflatoxins and other mycotoxins have been tabulated in Table 4. 

## 5. Conclusions and Future Prospective

This review paper has provided an insight on the analytical performance of fluorescence biosensor towards monitoring of mycotoxins. It was concluded that commonly used signal generating probes can be replaced by fluorescent labels as direct output signal generating probes, eliminating the problem of dyes. Bioreceptors can be modified with different fluorophores to perform the fluorescent-based detection. Fluorescence biosensors offer the advantages of short analysis time, cost effectiveness and ease in manipulation. However, only a single fluorophore molecule can be labelled with a bioreceptor, resulting in the decreased sensitivity of the system. Moreover, most of the commonly used fluorophores have fluorescent life times in seconds, and require specific storage conditions to stablize their fluorescent response. The decreased stability and short life time make these fluorescence-based assays expensive and unsuitable for onsite analysis. In this context, recent research may focus on the use of fluorescent nanoparticles as label probes in affinity-based assays to overcome the above described limitations. Similarly, with the advent of nanotechnology, various nanomaterials have been employed for the construction of fluorescence quenching-based assays for the detection of myctoxins. In this direction, future research can be performed on the modulation of surface chemistry of nanomaterials/nanocomposite to design target specific label free assays for the detection of various analytes.

## Figures and Tables

**Table 1:** Reports on fluorescence immunosensing platforms for OTA detection.

S. No.	Method/Principle	Matrix	Linearity (ng mL^−1^)	LOD (ng mL^−1^)	Reference
1.	Fluorescence polarization competitive immunoassay (FPIA)	Barley	5.0 × 10^3^–200.0 × 10^3^	3.0 × 10^3^	[36]
2.	Fluorescence	Coffee and wine	3.8–100	7–38	[37]
3.	Fluorescence	Corn	-	15	[38]
4.	FPIA	Wine	2.0–5.0	0.7	[39]
5.	Florescence resonance energy transfer (FRET)	Wheat	20–100	1	[40]
6.	Fluorescence	Wine and Corn	-	12 × 10^−4^	[41]
7.	MIPSE-FLD	Wheat	3–18	1.2	[42]
8.	Fluorescence coupled ELISA	Corn	0.0024–0.625	0.0022	[43]
9.	Fluorescence quenching	Wheat, corn and rice	5 × 10^−5^–1 × 10^−4^	5 × 10^−5^	[44]
10.	Fluorescence quenching	Wine and grape	0.08–5.0	0.06	[45]

OTA: ochratoxin A; MIPSE-FLD: Molecularly imprinted solid phase extraction coupled with fluorescence detector; LOD: Limit of detection.

**Table 2:** Reported literature based on fluorescence immunosensing platforms for mycotoxin other than OTA.

S. No.	Analyte	Method/Principle	Matrix	Linearity (ng mL^−1^)	LOD (ng mL^−1^)	Reference
1.	Aflatoxin M1 (AFM1)	Surface plasmon-enhanced fluorescence (SPFS)	Milk	10^−6^–1.0	6 × 10^−4^	[46]
2.	Aflatoxin B1 (AFB1)	Fluorescence (a) Flow set up (b) MTP (microtiter plate)	Peanut	0.5–7 0.5–30	0.2 0.1	[47]
3.	AFB1	FRET	Human serum	0.031–0.187	0.006	[48]
4.	AFB1	FRET	Rice	0.06–5	0.04	[49]
5.	AFB1	Time resolved fluorescence (TRF) based immunosensor	Peanut, corn, vegetable oil	0.2–60	0.06 to 0.12	[50]
6.	AFB1	Fluorescence polarization immunoassay (FPIA)	Beer sample	-	1	[51]
7.	AFM1	Total internal reflection fluorescence (TIRF)	-	0.073–0.400	0.045	[52]
8.	AFM1	Fluorescence	Milk	-	5	[53]
9.	Fumonisin B1 (FB1)	Fluorescence	Corn	10–1000	10	[54]
10.	Deoxynivalenol (DON)	Fluorescence	Corn	-	150	[38]
11.	Zearalenone (ZEN)	SAM and DAM-FLISA	Cereals	-	0.6 and 1.8	[57]
12.	FB1 and FB2	FPIA	Maize	-	157.4 (FB_1_) and 290.6 (FB_2_)	[56]

**Table 3:** Reported literature on fluorescence-based aptasensors for OTA detection.

S. No.	Principle/Material	Linearity (ng mL^−1^)	LOD (ng mL^−1^)	Matrix	Reference
1.	Graphene oxide—bare graphene PVP coated graphene oxide	8.01 × 10^−1^–14.133 × 10^−3^ 20.19–201.9	0.767 0.955	Beer	[60]
2.	Single-walled carbon nanotubes (SWCNTs)	10.09–80.76	9.73	Beer	[61]
3.	Structure switching aptamer assay	0.002–10	0.001	Corn	[62]
4.	Structure switching aptamer assay	1–100	0.8	Corn	[63]
5.	GNP-based FRET	0.005–5	0.002	Maize	[64]
6.	Terbium (Tb^3+^)	0.1–1	0.02	Wheat	[65]
7.	Nanographite (Amplified fluorescent aptasensor)	-	8.07	Red wine	[66]
8.	DNA-scaffolded silver-nanoclusters	0–30	0.002	Wheat	[67]
9.	Fluorescence	3.63–40.38	3.63	-	[68]
10.	Fluorescence (competitive assay) Fluorescence (re-suspended beads) Fluorescence (supernatant)	0.04–60.57 0.08–56.53 0.10–20.19	0.002 0.085 0.092	Beer	[69]
11.	Titanium dioxide nanoparticles (TiO_2_-NPs)	0.60–403.8	0.60	Beer	[70]
12.	TiO_2_-NPs	6.86–2020	0.55	Beer	[71]
13.	Cademium-telluirde (CdTe) QDs-MoS_2_ nanosheets	1–1000	1	Red wine	[19]
14.	Graphene quantum dots (GQDs)	0–1	0.013	Red wine	[72]
15.	Nitrogen doped carbon dots and silver nanoparticles	4.04–2020	3.53	Flour and beer	[73]

**Table 4:** Reported literature based on fluorescence aptasensing platforms for mycotoxins other than OTA.

S.No	Analyte	Principle	Matrix	Linearity (ng mL^−1^)	LOD (ng mL^−1^)	Reference
1.	AFB1	Fluorescence	Buffer peanut oil	0.99–6250 4.99 × 10^−1^–49.96 × 10^−4^	0.31 0.44	[75]
2.	AFM1	Fluorescence	Milk	0.001–2.0	0.005	[76]
3.	AFB1	Fluorescence	Beer and wine	0.25–32	0.2	[77]
4.	AFB1	Fluorescence	Peanut and corn	0.005–2.00	0.005	[29]
5.	AFB1, AFB2, AFG1, AFG2	Fluorescence	-	2.4–48	0.05	[78]
6.	AFB1	Fluorescence	Rice and peanut	3.12–124.91	1.06	[79]
7.	AFB1	Fluorescence	Infant Rice cereal	5–100	1.6	[80]
8.	AFB1	Fluorescence	Dried red chilies, groundnut, and whole pepper	0.05–50	0.01	[81]
9.	FB1	FRET	Maize	0.1–500	0.1	[20]
10.	ZEN	FRET	Beer and wine	0.5–64	0.5	[82]
